# Use of the reach, effectiveness, adoption, implementation, and maintenance (RE-AIM) framework to guide iterative adaptations: Applications, lessons learned, and future directions

**DOI:** 10.3389/frhs.2022.959565

**Published:** 2022-10-17

**Authors:** Russell E. Glasgow, Catherine Battaglia, Marina McCreight, Roman Ayele, Anna M. Maw, Meredith P. Fort, Jodi Summers Holtrop, Rebekah N. Gomes, Borsika Adrienn Rabin

**Affiliations:** ^1^Colorado Implementation Science Center for Cancer Control, Dissemination and Implementation Science Program, Adult and Child Center for Outcomes Research and Delivery Science, Department of Family Medicine, University of Colorado Anschutz Medical Campus, Aurora, CO, United States; ^2^Denver/Seattle Center of Innovation for Veteran-Centered and Value-Driven Care, Department of Veteran Affairs, VA Eastern Colorado Health Care System, University of Colorado Anschutz Medical Campus, Aurora, CO, United States; ^3^Division of Hospital Medicine, University of Colorado Anschutz Medical Campus, Aurora, CO, United States; ^4^Colorado Implementation Science Center for Cancer Control, Dissemination and Implementation Science Program, Adult and Child Center for Outcomes Research and Delivery Science, University of Colorado Anschutz Medical Campus, Aurora, CO, United States; ^5^Health Systems, Management and Policy Department, Colorado School of Public Health, University of Colorado Anschutz Medical Campus, Aurora, CO, United States; ^6^Dissemination and Implementation Science Center, Altman Clinical and Translational Research Center, Herbert Wertheim School of Public Health and Human Longevity in Science, University of California, San Diego, CA, United States

**Keywords:** adaptation, Iterative RE-AIM, partner engagement, PRISM, rapid research, implementation strategy, audit and feedback

## Abstract

**Introduction:**

Implementation science frameworks have been used widely for planning and evaluation, but seldom to guide adaptations during program implementation. There is great potential for these frameworks to be used to inform conceptual and data-driven decisions about adaptations.

**Methods:**

We summarize recent applications using Iterative RE-AIM to capture and guide adaptations. Iterative RE-AIM can be repeated at multiple time points customized to each project and involves the following activities: identification of key implementation partners; rating importance of and progress on each RE-AIM dimension (reach, effectiveness, adoption, implementation, and maintenance); use of summary data on ratings to identify one or two RE-AIM dimensions for adaptations and implementation strategies; and evaluation of progress and impact of adaptations. We summarize recent and ongoing Iterative RE-AIM applications across multiple care coordination and pain management projects within the Veterans Health Administration, a hypertension control trial in Guatemala, a hospital-based lung ultrasound implementation pilot, and a colorectal cancer screening program in underserved communities.

**Results:**

Iterative RE-AIM appears feasible, helpful, and broadly applicable across diverse health care issues, interventions, contexts, and populations. In general, the RE-AIM dimension showing the largest gap between importance and progress has been Reach. The dimensions most frequently selected for improvement have been Reach and Implementation. We discuss commonalities, differences and lessons learned across these various applications of Iterative RE-AIM. Challenges include having objective real time data on which to make decisions, having key implementation staff available for all assessments, and rapidly scoring and providing actionable feedback. We discuss print and online resources and materials to support Iterative RE-AIM.

**Conclusions:**

The use of Iterative RE-AIM to guide and support understanding of adaptations has proven feasible across diverse projects and in multiple case studies, but there are still questions about its strengths, limitations, essential components, efficiency, comparative effectiveness, and delivery details. Future directions include investigating the optimal frequency and timing for iterative applications; adding contextual assessments; developing more continuous and rapid data on which to make adaptation decisions; identifying opportunities to enhance health equity; and determining the level of facilitation that is most cost-effective.

## Introduction

There is emerging consensus among implementation scientists that adaptations to interventions and implementation strategies are inevitable, can be beneficial or detrimental, and need to be carefully documented and better understood (1–7). Implementation science theories and frameworks have been widely used to plan and tailor interventions and implementation strategies (8–10), and some models such as the Implementation Outcomes Framework (IOF) (11) and the Reach, Effectiveness, Adoption, Implementation, and Maintenance (RE-AIM) (12) framework to guide outcomes evaluation. However, there has been little iterative use of these frameworks during the implementation phase or to guide adaptations (13). While Kirk et al. have developed the Model for Adaptation Design and Impact (MADI) (14) to comprehensively characterize adaptations and how they impact outcomes and general guidance has been provided in ADAPT-ITT (15), very few empirical studies have evaluated the actual impact of adaptations qualitatively or quantitatively (16–18). The ADAPT (7) guidance explicitly calls for continuous iterative review of emerging adaptations but did not identify tools or resources for doing this. Below we describe Iterative RE-AIM that shares many similarities with these three approaches but is distinct because of its specific and comprehensive use of an implementation science outcomes framework (RE-AIM); focus on rapid repeated adaptations; and provision of specific directions, tools, survey items and feedback displays.

Real world application of complex health interventions (19, 20) in complex, dynamic systems guarantees that a program will seldom be implemented in diverse non-research settings exactly as planned, no matter how comprehensive and well done the planning. This is especially true of interventions that prescribe specific actions as in a detailed protocol, although likely less so for interventions providing more general guidelines (21) or key “functions” for the intervention (22). Thus, adaptations during implementation are ubiquitous and it would be advantageous if the adaptations could be guided by a conceptual framework and based on data rather than guess work. Our group has published initial work using the RE-AIM framework to guide adaptations (13).

For background, RE-AIM, and its more recent expansion into the Practical Robust Implementation and Sustainability Framework (PRISM), are implementation science frameworks that focus on key outcomes necessary to produce population impact and contextual factors that influence these outcomes. RE-AIM (13, 23) is one of the most widely used implementation science frameworks to assess implementation outcomes and PRISM, which includes RE-AIM dimensions, adds a focus on key contextual factors related to these outcomes. The PRISM domains include individual and organization/setting characteristics; individual and organization/setting perceptions of the intervention; external environmental factors (e.g., relevant policies, reimbursement issues, community influences); and implementation and sustainability infrastructure (e.g., resources, job responsibilities, and processes to support implementation).

In this paper, we present Iterative RE-AIM as a method for assessing progress toward goals set for RE-AIM outcomes, prioritizing areas needing improvement, and identifying adaptation strategies for these areas. Iterative RE-AIM then monitors improvements following these changes. The process is undertaken on multiple occasions (i.e., iteratively) to continue refining intervention delivery. The methods and key functions of Iterative RE-AIM are described in the Methods and results section and Table 1 but in brief, it provides a concrete, structured way to engage implementation team members and to foster discussions of both progress to date and current priorities using RE-AIM as a framework. Team members individually complete a brief survey about their perceptions of both progress and their priorities across the five RE-AIM dimensions of reach, effectiveness, adoption, implementation, and maintenance at that point in time. Results are then integrated and summarized for discussion at an upcoming meeting. These discussions are based on a “gap analysis” of the dimensions on which there is the greatest difference between priority and progress to develop consensus strategies for adaptations to enhance progress on one or two key RE-AIM areas. This process is then repeated at a frequency tailored to the given project.

**Table 1:** Key functions of iterative RE-AIM.

**Key functions of Iterative RE-AIM**
-Education on key issues and dimensions in RE-AIM (or PRISM) so team members have a conceptual understanding of and can utilize the RE-AIM dimensions to set priorities and evaluate progress
-Obtain independent input and perspectives from each team member; then summarize results in visual displays
-Team analyzes, reflects on, and discusses progress and priorities at that time point;
-Specify 1–2 team RE-AIM priority areas and adaptations for next implementation period
-Implement and evaluate the delivery and impact of adaptations
-Learn from iterations and repeat as appropriate over time

Based on encouraging results from initial application of Iterative RE-AIM across multiple projects in a Veteran's Administration Quality Enhancement Research Implementation Initiative (13), we have recently used or are using Iterative RE-AIM in several additional projects. The purposes of this paper are to: summarize four recent and ongoing applications of Iterative RE-AIM to guide adaptations; describe key findings and lessons learned in these applications; and identify directions for future research and practice using Iterative RE-AIM to assess and guide adaptations.

## Methods and results

### Iterative RE-AIM functions, process and resources

#### Iterative RE-AIM key functions

There was some variation in the specific activities and processes used across the case studies in this paper. However, as shown in Table 1, across projects there are several key functions of Iterative RE-AIM that were accomplished in different forms in different projects.

#### Iterative RE-AIM process

To accomplish the key functions described in Table 1, Iterative RE-AIM is conducted using the steps and processes described below. Table 2 summarizes the key steps, implementation strategies, and activities involved in Iterative RE-AIM. The key steps are: (1) At an initial meeting with the identified implementation team members there is a general overview of the Iterative RE-AIM process, review of pragmatic RE-AIM definitions and how they are operationalized, and general discussion of the program or evidence-base practice (EBP) involved; (2) Team members each provide a confidential rating on the importance (group member perceptions) of and progress on all RE-AIM dimensions (using actual data when possible) and results are analyzed and summarized in a way that protects the identify of individuals; (3) The team reviews, reflects on and discusses the ratings using visual displays that summarize ratings; (4) The team identifies one or two RE-AIM dimensions on which to focus, and identifies adaptations (implementation strategies) to address these areas; (5) The agreed upon adaptations and implementation strategies are implemented and short term impact is evaluated; and (6) Future meetings are held approximately every 1–2 months, repeating steps 1–5, which allows for changes in goals and implementation strategy adaptations based on progress.

**Table 2:** Steps and component activities in applying Iterative RE-AIM.

**Step and activity in the Iterative RE-AIM process**	**Key implementation strategies, description, and examples**
1.Identifiation of team members and education about RE-AIM (PRISM) and Iterative RE-AIM	Education and training: basics of RE-AIM (PRISM) using slides, animated video, and discussion
2. Anonymous completion of survey on RE-AIM (PRISM) dimensions	Audit; monitoring: Team members provide independent ratings of priority of and progress on all RE-AIM (PRISM) dimensions
3. Presentation and discussion of results	Reflection; Consensus Building: Review and discuss results using “Gap Analysis” feedback display
4. Structured discussion and priority setting; Identification of adaptations	Facilitation; goal setting; and action planning: Brainstorm, estimate feasibility and impact, revise and commit to new adaptations (new or additional implementation strategies)
5. Implementation of planned adaptations; Evaluation of impact	Audit and feedback: Evaluate strategy implementation and proximal results on RE-AIM outcomes
6. Repeat the above	Audit and feedback: Review and act on longitudinal data, decide upon frequency and timing of Iterative RE-AIM to fit project and progress

#### Iterative RE-AIM resources

To facilitate conduct of Iterative RE-AIM, several key resources are available. These materials are publicly available at https://re-aim.org/applying-the-re-aim-framework/re-aim-guidance/use-during-implementation/ and include:

- Introductory and educational Iterative RE-AIM materials to orient team members to the concepts in RE-AIM and outline the process used;
- Iterative RE-AIM worksheets containing brief survey questions for team members to record their scores on progress and importance;
- Iterative RE-AIM gap analysis tool (in Excel) to calculate group scores and develop summary reports;
- Sample visual displays of results of the above ratings;
- Action planning forms to provide a written record of the adaptations and implementation strategies planned;
- An Iterative RE-AIM evaluation form to assess the usefulness and impact of the Iterative RE-AIM process.

### Case studies

This article is not a standard quantitative report of a trial nor an in-depth qualitative study. Rather it is a compilation of results, experiences and lessons learned across a variety of different applications of Iterative RE-AIM. Thus, instead of following a traditional reporting system or results section, we have organized each case report using the headings of: Description of program and use of Iterative RE-AIM; and Findings. This is summarized in Table 3. This is followed by a section on Crosscutting Lessons Learned.

**Table 3:** Summary of current and ongoing Iterative RE-AIM projects.

**Project/setting: Guatemala (** **24** **–** **26** **)**	
Health topic	Hypertension control
Team members involved (# and type)	- Ministry of Health staff - Research project staff: 3 MDs, local-level evaluators
Number of iterations	Quarterly for Implementation (which was the primary focus)
RE-AIM dimensions most frequently selected	Implementation and Context (relevant to PRISM)
Key adaptations and implementation strategies	- Monitoring of availability of 3 HTN meds, BP monitor, provider turnover - Assessment of Implementation (e.g., coaching sessions, team-based care, training) - Adaptations during COVID-19 (delivery of meds to patients' homes, hybrid vs. in-person training)
**Project/setting: Hospital**	
Health topic	Point of care lung ultrasound (LUS)
Team members involved (# and type)	−4 hospitalist implementors - 12 hospitalists eligible for adoption - 2 hospitalist clinical leaders
Number of iterations	24: Twice monthly over a period of 12 months
RE-AIM dimensions most frequently selected	Reach and adoption
Key adaptations and implementation strategies	- Evaluation of LUS dashboard based on real time EHR data - Employ data from clinician interviews to understand barriers to adoption - Deployment of new strategies based on qualitative and quantitative data - De-implement strategies that were not working
**Project/setting: Multiple VA and community settings**	
Health topic	Care coordination and pain management
Team members involved (# and type)	There are site champions for each EBP at each VA site. Depending on the EBP, team members were quite varied and included leadership, community partners, and Veterans.
Number of iterations	Baseline assessment and periodic assessments every 4–6 months at each site
RE-AIM dimensions most frequently selected	TBD
Key adaptations and implementation strategies	TBD
**Project/setting: Accelerating colorectal cancer screening using implementation science (ACCSIS) San Diego**	
Health topic	Colorectal cancer screening
Team members involved (# and type)	2 Federally Qualified Healthcare Centers (FQHC), hub organization representative, research team (separate sessions for each health center, number of participants range from 7 to 14 with increased number of health center representatives attending the mid-implementation assessment)
Number of iterations	Two: Pre-implementation and mid- implementation Use and discussion of PRISM context survey items at both time points
RE-AIM dimensions most frequently selected	Data from pre-/mid- implementation assessments (implementation ones just completed and in progress of analysis): - Overall high ratings on most dimensions - Overall, relatively lower ratings on abnormal FIT follow-up compared to mailed FIT - Overall lower ratings during the mid-implementation vs. pre-implementation - Variation across the health centers on lower areas - “Adoption implementer” dimension is not seen as relevant by multiple participants (i.e., it is not a choice of individual adopters to participate) - Pattern of lower ratings for Implementation— (especially cost and resources), Reach (only includes those with insurance), and Maintenance (need for ongoing support to undertake both mailed FIT delivery and Abnormal FIT follow-up) - Lower alignment scores for the following PRISM context domains: Implementation and sustainability infrastructure, Recipient characteristics—organizational, and External environment
Key adaptations and implementation strategies	TBD—currently in progress

#### Hypertension control in Guatemala

##### Description of the program and use of Iterative RE-AIM

During a hypertension control study in 5 departments (provinces) and 36 districts of Guatemala, we used PRISM/RE-AIM for planning and evaluation and assessed dimensions and aspects of context at multiple time points (24). As described below PRISM, the Practical Robust Implementation and Sustainability Model (23) focuses on key contextual factors related to RE-AIM outcomes. We recognized from the outset that it would be important to prioritize the assessment of delivery of five implementation strategies and aspects of context regularly during the 18-month study. Prior to implementation we conducted a needs assessment (25) in which we identified routine assessment of availability of medications in health posts/centers as a top priority to review at monthly meetings throughout the study. The implementation phase of the study began in 3 provinces in the Eastern part of the country and subsequently in 2 provinces in the Western Highlands. We developed implementation tracking forms (for the RE-AIM Implementation domain) that were filled out by implementers (Ministry of Health staff, primarily auxiliary nurses within intervention districts). Local-level project evaluators, assigned to cover two districts each, captured data using forms to assess key aspects of context within health posts and centers (availability of medications, blood pressure monitors, and staff turnover). The team met regularly with the Ministry of Health at the central level to be aware of broader contextual changes (e.g., service priorities or trainings that would influence providers' time and availability). At monthly research meetings, we reviewed and reflected on changes in Implementation and medication availability and discussed staff turnover and implications for the PRISM contextual factor of Implementation and Sustainability Infrastructure. We reviewed Reach during initial meetings but decided that it would be difficult to influence that dimension in the short-term even though men were participating at a much lower rate than women. The COVID-19 pandemic began during the rollout of the trial. This resulted in a dramatic change to the context, and the study team and Ministry of Health staff identified the need for major adaptations. Some of the key adaptations that we made were: a change in how training was conducted (from in-person to hybrid) and increased flexibility in providing medications to patients (more than 1-month supply, allowing family members to pick up medications, shifting medications from health centers to posts to cut down on distance that patients needed to travel).

##### Findings

Due to the large number of sites and long distances between them (anticipated) and disruption during the COVID-19 pandemic (unanticipated), we recognized how important it was to have a system in place to track Implementation and contextual issues. The study team took time to review and reflect on data during monthly meetings, using PRISM/RE-AIM, and we discussed key areas on which to follow up—this usually led to reaching out to different actors in the Ministry of Health at the central, provincial, or local levels. Medication availability and staff turnover were recognized as key. Early during the pandemic, when it was not possible to travel, the team felt disconnected from what was happening in the rural communities many hours from the capital city; the project staff decided to make phone calls to implementers and patients to gain insight into their experiences (26) and to inform adaptations. The qualitative and quantitative data obtained during those phone calls helped the team define next steps at a critical moment of uncertainty.

#### Hospital based point of care lung ultrasound

##### Description of the program and use of Iterative RE-AIM

Our 12-month long lung ultrasound implementation pilot was conducted at an academic quaternary care medical center in Aurora, Colorado in response to the COVID pandemic (27). The goal of the pilot was to quickly implement the use of lung ultrasound among hospitalist clinicians caring for adults hospitalized with COVID to conserve personal protective equipment and reduce COVID exposure within the hospital environment caused by use of chest imaging modalities performed by radiology. Iterative RE-AIM was the overarching implementation strategy used in this pilot study. Given the low baseline rate of lung ultrasound use among hospitalists at the beginning of the pilot, the implementation team chose to prioritize the RE-AIM outcome measures of Reach and Adoption. To iteratively measure Reach and Adoption, a novel RE-AIM dashboard was created to display these quantitative measures using data extracted from the EHR and automatically updated every 48 h. While the dashboard took some resources and expertise to build, it required minimal time to use and maintain over the course of the study, providing nearly real-time access to these prioritized implementation outcomes. At twice monthly meetings, updated RE-AIM dashboard data were evaluated and discussed by the implementation team which consisted of 4 hospitalists, to screen for barriers to Adoption and Reach. Qualitative data were collected concurrently through interviews with hospitalist faculty to understand contextual factors and determinants of adoption. Through discussion of this qualitative and quantitative data, the implementation team would come to consensus regarding interval adaptations to on-going implementation strategies.

##### Findings

Through this project we learned that operational dashboards make iterative assessment of RE-AIM outcomes drawn from EHR data highly feasible, allowing for easy monitoring of both the progress and representativeness of some RE-AIM dimensions and data-driven interim adjustments in implementation strategies. Future work will focus on more formally and systematically incorporating rapid qualitative methods (28) guided by the contextual domains of PRISM into our Iterative RE-AIM process to better understand current barriers to implementation detected by iterative evaluation of data displayed *via* a RE-AIM dashboard.

#### Colorectal cancer screening project integrating assessment of PRISM contextual factors

##### Description of the program and use of Iterative RE-AIM

In this NCI-funded research project focusing on increasing colorectal cancer screening in underserved communities in San Diego County, our team works with a bridge organization and two federally qualified healthcare systems (FQHCs) using a hub (i.e., bridge organization) and spoke (i.e., FQHCs) model as an implementation strategy to increase the completion of colorectal cancer screening and follow up of abnormal screening results. We are using PRISM and a PRISM Fit Assessment at two time points, pre- and mid- implementation with each FQHC separately. At both stages, a survey instrument was administered using REDCap to capture perceptions of representatives of the FQHCs, the bridge organization, and the research team on how likely it is that reach, adoption, implementation, effectiveness, and maintenance of the research program will be optimal. The survey also asked whether the program aligned well with PRISM contextual domains of: perceptions of the diverse partners and patients of the program, characteristics of these diverse partners, the implementation and sustainability infrastructure at their health care center, and the external environment. Data from this survey from multiple participants were summarized in visual displays and summary points and shared during a follow-up meeting including all who completed the survey. The meeting allowed for discussion of areas that scored low consistently, reasons for low scores, and possible adaptations to improve these areas. Key discussion points and related action steps were summarized and shared with each FQHC along with a cross-FQHC summary.

##### Findings

Data from pre- and mid- implementation assessments indicated that most RE-AIM dimensions and PRISM domains were rated relatively highly on all dimensions. Lower ratings were noted for abnormal FIT follow-up compared to the mailed FIT intervention and for the mid-implementation assessment compared to the pre-implementation assessment. Variation was noted across the health care centers in areas of lower ratings. Some areas like adoption—at the implementer level—were not deemed meaningful because staff at the participating FQHCs did not have a choice of opting out of the program. To address this concern, a “not applicable” answer option was added to the survey questions. It was also indicated that the distinction between general patient population and underserved populations in terms of reach, effectiveness, and maintenance was not as relevant as the FQHCs exclusively serve underserved communities. However, it was noted that the program reach was somewhat limited by only including individuals with health insurance. Key RE-AIM dimensions with lower scores were Reach, Implementation (especially as it relates to cost and resources), and Maintenance. Some PRISM context domains with lower alignment scores included Implementation and Sustainability Infrastructure, Recipient Characteristics—organizational, and External Environment. The follow-up sessions when results were discussed allowed for rich discussions between the research team and implementation partners. Key themes identified included the need to consider sustainment, costs and resources needed to deliver the intervention after the study is completed, strategies to reach patients with no insurance, and the external environment including possible policy impact.

#### Quadruple aim quality enhancement research initiative (QUERI)

##### Description of the program and use of Iterative RE-AIM

The goal of the Department of Veterans Affairs (VA) Quadruple Aim QUERI is to enhance Veteran outcomes and experiences, clinician engagement, and reduce the cost of care by providing value-based care coordination between VA and community settings for Veterans and implementation partners using sustainable practices. Veterans who receive care in both VA and community settings (dual-use) are at risk for fragmented, poorly coordinated care across care settings, which may contribute to adverse outcomes, poor experiences, and increased costs of care (29, 30).

We are implementing three evidence-based practices (EBPs), one in each of three different health services projects, all rooted in care coordination models to achieve consistently safe, efficient and effective care for Veterans. These three EBPs offer care coordination programs throughout the continuum of care to facilitate the integration of, and navigation through, healthcare services within and across care settings, to help patients receive the care they need and want without unnecessary duplication of services or unwarranted delay (31).

We are using two evidence-based implementation strategy bundles to guide EBP implementation: Iterative RE-AIM and Relational Facilitation (32), which were developed and tested in our previous work of the Triple Aim QUERI and the Office of Rural Health Rural Transition Nurse Program (33). As with these previous programs (6), we are assessing and guiding implementation and adaptations based on emerging data and changing context through the lens of PRISM (34) while addressing factors impacting the RE-AIM outcomes (13). Both Iterative RE-AIM and Relational Facilitation implementation strategy bundles include a set of transactional (i.e., training, audit and feedback) and transformational (i.e., goal setting, strengthening and sustaining team relationships) strategies.

During **pre-Implementation**, both implementation strategy bundles are being employed with PRISM to engage multi-level partners and identify relevant contextual factors. Relational Facilitation is being used to assess, guide, and develop high-quality interprofessional cross-setting relationships for the purpose of task integration. Relational Facilitation strategies are being implemented in partnership with clinical intervention leads and adapted based on each site's needs. PRISM is being used repeatedly to inform adaptions so they align with context, beginning with the pre-implementation phase.

Currently, we are i**mplementing** both implementation strategy bundles to support the teams to review implementation data and rate progress on RE-AIM outcomes and then reflect on the “gap” between rated importance and their progress. Iterative RE-AIM assessments will guide adaptations and action plans, especially by using evidence to direct efficient decisions about care approaches. Progress at each assessment will guide resource allocation and intensity of Relational Facilitation for the subsequent period. The two implementation bundles support each other and are designed to begin with the lowest intensity of facilitation activities using the “minimal intervention needed for change” approach (35). Based on the results of iterative assessments, progress on outcome measures and priorities of the EBP teams, more intensive activities will be applied in an iterative manner, while tracking time and costs.

##### Findings

We have faced some challenges as we rolled-out each implementation strategy bundle across the different EBPs at various VA sites. We rolled out Relational Facilitation and Iterative RE-AIM separately, in that order, to minimize the staff burden at the local site, and it remains to be seen what impact this will have. We have found it necessary to adapt the process we had used in our previous research with Iterative RE-AIM to work with implementation partners who have less time and engagement, assess the site's priorities, define, and operationalize RE-AIM outcomes as well as adapt requirements for staff training due to our remote work environment.

#### Crosscutting lessons learned

Although there were differences across the case studies that utilized Iterative RE-AIM, there were also crosscutting findings that emerged across projects. Table 4 and the text below summarize these findings.

**Table 4:** Key crosscutting issues in applying Iterative RE-AIM.

**Issue**	**Description**
Implementation partner engagement	Advantageous to get persons that will be impacted from different perspectives involved- e.g., organizational decision makers, clinicians, front line delivery staff, recipients (patients); however, getting a large number and variety of people to consistently attend meetings can be challenging
Data for decision making	It is challenging to get rapid reliable data rather than perceptions on RE-AIM outcomes and then to display results in ways that are clear and actionable. Project records can be designed and automated to make it easier to obtain data for issues such as reach, and implementation issues such as fidelity and adaptations
Discussion of progress and priorities	The core issue and “secret to success” of Iterative RE-AIM is sharing and discussing both the objective and subjective data and perceptions; discussing similarities and differences of opinion (and reasons why); and coming up with consensus strategies for action
Evaluating impact	While not unique to Iterative RE-AIM, in most projects it is difficult to attribute changes in intermediate outcomes to action plans and Iterative RE-AIM based adaptations implemented due to many changing variables, lack of experimental design, and miscellaneous uncontrolled factors,
Time and resources required	One needs to decide how much time and resources to devote to Iterative RE-AIM. This can range from minimal- doing it once at middle of program, using whatever staff are available, and relying solely on staff perceptions for goal setting/strategy selection- to several systematic iterations involving comprehensive data collection and detailed adaptation tracking
Balancing standardization and adaptation	It is necessary to strike a balance between accomplishing the key functions of Iterative RE-AIM in Table 1 and making appropriate adaptation to the forms needed in different settings and contexts having different data sources, resources, and priorities

##### Finding #1: Engagement of the persons implementing, making decisions about, or impacted by a program is both important and challenging

The case studies employed different numbers and types of clinical and community partners, but most often centered on the team directly implementing the program. It is important to have team members share perceptions and agree on priorities, but it is unclear how many perspectives are needed and if these need to be the same persons across all Iterative RE-AIM assessments. Although most recommendations regarding team science (36) stress including a full array recipients (e.g., patients, employees, opinion leaders, organizational decision makers, community representatives) as part of the decision making team, the example cases did not involve all these categories of partners. Congruent with recent emphases and recommendations for complex interventions (20) and adaptations (7) we are finding the level of engagement of multiple implementation partners to be critical for success. However, including a larger number and different types of participants needs to be balanced against the logistics and costs of those members being able to meet regularly to continue the Iterative RE-AIM process over time. It will be informative to see if Iterative RE-AIM applications that involve more partners with more diverse perspectives produce better long-term results than those that do not.

##### Finding #2: Having real time objective data on RE-AIM outcome for use to evaluate progress is ideal but challenging

**Figure 1:**
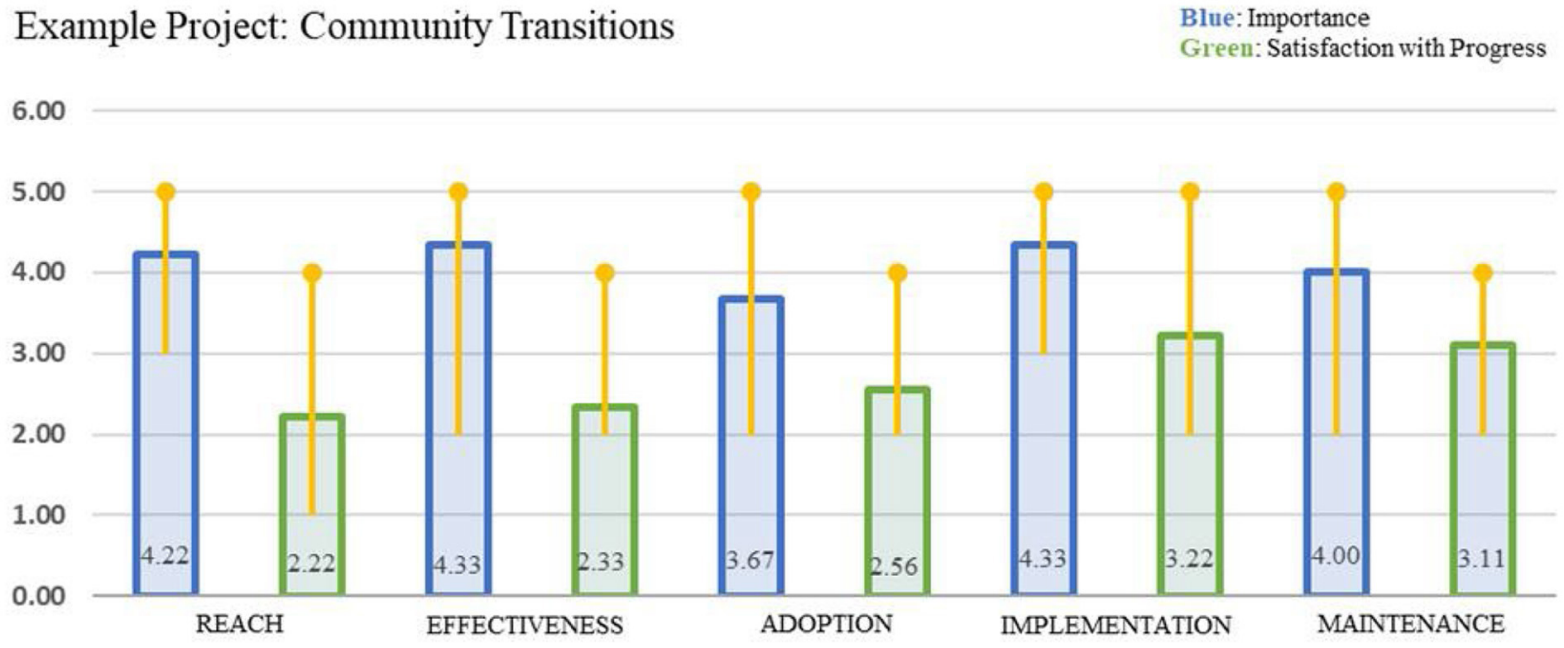
Example of Iterative RE-AIM gap analysis to study discrepancies between importance and progress on RE-AIM outcome dimensions.

Except for the lung ultrasound study, the current Iterative RE-AIM applications did not have real time, objective data on RE-AIM outcomes to evaluate progress. Sometimes project records provided information on Reach or Adoption rates, but many of the ratings of progress were made based on the subjective impressions of the team members. Design and proactive use of process data systems that can be queried to produce frequent updates on issues such as fidelity, adaptations, and representativeness (equity) of RE-AIM results would improve the quality of data available for decision making. Once data on progress on RE-AIM dimensions are available, they need to be summarized and communicated in a way that is readily understood and actionable. Current Iterative RE-AIM projects have used some form of a bar chart as shown in Figure 1, and most participants seem to understand and find these displays useful, with exception that information about variability across raters was unclear for some participants. Newer applications of Iterative RE-AIM are experimenting with different types of visual displays, including giving participants their choice of different data displays.

##### Finding #3: Leaders of all the Iterative RE-AIM projects agreed that the implementation team exchanging perspectives on progress and priorities, and then making a group decision about the most appropriate area(s) to target and strategies to use is the core of the Iterative RE-AIM process

These discussions can be rich and enlightening for participants but can also require experienced facilitation if there are large differences in perception, power, or information across team members.

##### Finding #4: Evaluating the impact of the iterative adaptations selected is challenging and has been suboptimal to date

The primary method to date has been estimating progress at the next meeting across RE-AIM dimensions, but this is non-specific and suffers from the same concerns about data quality noted above. Even with high quality data, without experimentally testing strategies, it is difficult to attribute improvement to use of a strategy separate from numerous other dynamic program and contextual factors (19, 37). This is a conceptual and methodological challenge for all approaches to adaptions, not just Iterative RE-AIM. Since it is impossible in many situations such as our Quadruple Aim QUERI project to separate and independently evaluate the impact of separate implementation strategies, this may never be knowable. It is likely best addressed through mixed methods approaches using proximal quantitative data (such as rapid EHR data) followed by qualitative probes to provide confirmation and contextual understanding.

##### Finding #5: The amount of time and resources required for Iterative RE-AIM can vary considerably

The case studies vary from a single midpoint use of Iterative RE-AIM to numerous biweekly applications; the number of team members from one or two up to 14; and the work required to prepare data summaries from being very little when automated EHR reports are available to fairly time consuming if ratings from several persons need to be analyzed, integrated and feedback displays produced by hand.

##### Finding #6: There needs to be a balance between standardization of Iterative RE-AIM processes and adaptation to specific projects and contexts

We find useful the concepts of form and function (22) of adherence to core *functions* of Iterative RE-AIM as outlined in Table 1, while encouraging tailoring of the specific *forms*- e.g., data sources, data display choices, which staff to involve, number of iterations. We also experienced some challenges in making decisions about what constitutes an adaptation vs. just a small change that is not intended to improve fit to context.

## Discussion and future directions for practice and research

Our findings provide initial results from multiple projects utilizing a relatively new Iterative RE-AIM process to identify priorities, iteratively guide adaptations, and monitor progress over time. In general, use of Iterative RE-AIM was feasible for the projects to implement. The Iterative RE-AIM process revealed new insights for the team so that they could better discern how they wanted their implementation to proceed and what was most important through prioritizing specific RE-AIM dimensions. Progress over time was built into the process through the repeated Iterative RE-AIM cycles which specifically called out how the project was proceeding by gathering both data and perceptions. Just having the structure provided a way for the goals of a program to stay more present in the minds of the implementation teams.

Although the future use of Iterative RE-AIM is promising, there is still much to learn to maximize its effectiveness and efficiency. In this section, we key issues for practice of Iterative RE-AIM and identify opportunities for future research evaluating Iterative RE-AIM (Table 5). **First**, data availability to inform implementation actions and potential adaptations is important. We anticipate greater availability of EHR based dashboards such as that in our lung ultrasound project as well as close to real time data from ecological momentary assessments in the future. More systematic process data collection and tracking fields in project records on reach, equity, and implementation issues would also be helpful and should be feasible for most projects. Once data are collected, they need to be made available rapidly in easily understood and actionable formats. There is an opportunity to identify innovative ways to display data in visual dashboards and inform high quality data generation for rapid research and adaptations.

**Table 5:** Directions for future research and practice.

**Issue**	**Practice recommendation**	**Research need and opportunity**
Data availability and interpretation	Find low burden methods for data collection and display; ensure all staff understand the RE-AIM concepts	Investigate innovative ways to display data in dashboards and other visuals; develop high quality data for rapid research and ways to efficiently educate implementation team members on PRISM and RE-AIM
Capturing impact	Assure that both specific measures and the Iterative RE-AIM priorities selected reflect issues of greatest value to your system	Develop and validate pragmatic measures to rapidly assess impact of resulting strategies and adaptations
Capturing relevant aspects of context	Develop guidance for adapting Iterative RE-AIM to context; aligning with local history, resources, relationships, and workflow	Identify key aspects of context to consider in developing Iterative RE-AIM strategies using PRISM or other models. Employ rapid qualitative methods to assess and inform adaptations to align with local and dynamic context
Intensity of Iterative RE-AIM	Consider stepped care or minimal intervention needed for change (30) approach to see how many iterations are needed	Conduct cost-effectiveness and comparative effectiveness studies of different levels of facilitation, number and type of implementation partners involved, and frequency of Iterative RE-AIM
Equity implications	Review implications for equity at different time points and across PRISM/RE-AIM factors	Define dimensions of equity to consider across projects; support development of equity assessment as a core process within the implementation and sustainability infrastructure

**Second**, there is a need to more systematically assess impact and ensure that both specific measures and Iterative RE-AIM priorities reflect issues of the greatest value to the participants and system where the program is being implemented. This could be done by engaging implementation partners in the selection, refinement and use of pragmatic measures to rapidly assess the impact of resulting strategies and adaptations. **Third**, capturing relevant context to inform program implementation and adaptation should facilitate success. This could be operationalized by developing more explicit guidance for adapting Iterative RE-AIM to context and creating alignment with implementing system resources and relationships. Future research could identify key aspects of context for Iterative RE-AIM using PRISM or other conceptual models. Additionally, rapid qualitative methods (28), used in conjunction with “Iterative PRISM,” have the potential to serve as a powerful tool to acquire timely and actionable information on dynamic context. The information produced should allow for a better understanding of current barriers to RE-AIM outcomes and be used to adapt implementation strategies more effectively. These two innovative methods, both focused on improving the rapidity of implementation research should be explored. **Fourth**, the intensity and timing of Iterative RE-AIM need to be better understood. Practice applications of Iterative RE-AIM could consider stepped care or minimal intervention needed for change approaches (35). Future research could conduct cost effectiveness and comparative effectiveness studies of different levels of facilitation, which parties are involved, and the timing and frequency of Iterative RE-AIM. Finally, careful tracking and investigation of the impact of Iterative RE-AIM on health equity is needed, assessing both intended and potential unintended consequences.

Our findings should be interpreted in the context from which they were derived. Limitations to this report include that none of the case studies are experimental studies or compare Iterative RE-AIM to other implementation strategies. Also, only two of the case examples are completed projects and the ultimate impact of Iterative RE-AIM and the adaptions conducted is yet unknown. While two of the projects were directed and implemented by researchers other than the original developers of Iterative RE-AIM, all were led by investigators experienced in using RE-AIM. Future research should evaluate the level of expertise (in RE-AIM, implementation science, and group facilitation) required for successful use of Iterative RE-AIM. Strengths of this report include pragmatic use of Iterative RE-AIM across several projects diverse in terms of health care issues, settings, patient and staff characteristics and different forms of Iterative RE-AIM. Iterative RE-AIM appears to be broadly applicable, including during the rapidly changing and challenging context of the COVID-19 pandemic. We hope that by providing details of these different applications of Iterative RE-AIM and making resources to conduct Iterative RE-AIM publicly available https://re-aim.org/applying-the-re-aim-framework/re-aim-guidance/use-during-implementation/ will facilitate replication and investigation of its impact and usefulness in guiding adaptations across a variety of different conditions and contexts. We look forward to hopefully having enough applications of Iterative RE-AIM to conduct a more formal review in the future.

## Data availability statement

The raw data supporting the conclusions of this article will be made available by the authors, without undue reservation.

## Ethics statement

The studies involving human participants were reviewed and approved by COMIRB. Written informed consent for participation was not required for this study in accordance with the national legislation and the institutional requirements.

## Author contributions

REG and BR conceptualized the Iterative RE-AIM process. CB, MM, and RA developed most of the original materials, methods, and resources. RNG drafted the article and contributed to paper compilation. CB, MM, BR, AM, MF, and JH contributed case study examples. RA, BR, and MM contributed to the data collection and analysis. All authors contributed to the implementation of the projects, interpretation of results, and manuscript revisions.

## Funding

The research reported in this publication was supported in part by the National Cancer Institute of the National Institutes of Health (NIH) under Center P50 Grant Award Number: 5P50CA244688. It was also supported in part by the National Institutes of Health Grant Award Numbers: 5UH3CA233314-03 and UL1 TR002535. Also supporting this research in part was the National Heart, Lung, and Blood Institute Grant Award Numbers: K12 HL137862, U01HL138647, and 5K12HL137862. This work was also funded in part by the VA HSRD/QUERI.

## Conflict of interest

The authors declare that the research was conducted in the absence of any commercial or financial relationships that could be construed as a potential conflict of interest.

## Publisher's note

All claims expressed in this article are solely those of the authors and do not necessarily represent those of their affiliated organizations, or those of the publisher, the editors and the reviewers. Any product that may be evaluated in this article, or claim that may be made by its manufacturer, is not guaranteed or endorsed by the publisher.

## Author disclaimer

The contents of this work are the authors' sole responsibility and do not necessarily represent the official views of the VA.

## Abbreviations

RE-AIM: Reach, Effectiveness, Adoption, Implementationand Maintenance
PRISM: Practical Robust Implementation and Sustainability Infrastructure.

## References

[1] Chambers DA Norton WE. The adaptome: advancing the science of intervention adaptation. Am J Prev Med. (2016) 51(4 Suppl. 2):S124–31. 10.1016/j.amepre.2016.05.011 27371105 PMC5030159

[2] Stirman SW Miller CJ Toder K Calloway A. Development of a framework and coding system for modifications and adaptations of evidence-based interventions. Implement Sci. (2013) 8:65. 10.1186/1748-5908-8-65 23758995 PMC3686699

[3] Tabak RG Hook M Chambers DA Brownson RC. The conceptual basis for dissemination and implementation research. In: Brownson RC, Colditz GA, Proctor EK, editor. Dissemination Implementation Research in Health: Translating Science to Practice 2R. 2nd ed. New York, NY: Oxford University Press (2018). p. 73–88.

[4] Tabak RG Chambers D Khoong EC Brownson RC. Models in dissemination and implementation research: useful tools in public health services and systems research. Front Public Health Serv Syst Res. (2013) 2. 10.13023/FPHSSR.0201.0825558439

[5] Powell BJ Beidas RS Lewis CC Aarons GA McMillen JC Proctor EK. Methods to improve the selection and tailoring of implementation strategies. J Behav Health Serv Res. (2015) 44:177–94. 10.1007/s11414-015-9475-6 26289563 PMC4761530

[6] Rabin BA McCreight M Battaglia C Ayele R Burke RE Hess PL. Systematic, multimethod assessment of adaptations across four diverse health systems interventions. Front Public Health. (2018) 6:102. 10.3389/fpubh.2018.00102 29686983 PMC5900443

[7] Moore G Campbell M Copeland L Craig P Movsisyan A Hoddinott P. Adapting interventions to new contexts-the ADAPT guidance. BMJ. (2021) 374:n1679. 10.1136/bmj.n1679 34344699 PMC8329746

[8] Birken SA Powell BJ Shea CM Haines ER Alexis Kirk M Leeman J. Criteria for selecting implementation science theories and frameworks: results from an international survey. Implement Sci. (2017) 12:124. 10.1186/s13012-017-0656-y 29084566 PMC5663064

[9] Strifler L Cardoso R McGowan J Cogo E Nincic V Khan PA. Scoping review identifies significant number of knowledge translation theories, models, and frameworks with limited use. J Clin Epidemiol. (2018) 100:92–102. 10.1016/j.jclinepi.2018.04.008 29660481

[10] Rabin B Glasgow R. Dissemination & Implementation Models in Health Research Practice. University of California San Diego, University of Colorado Anshcutz Medical Campus (2022). Available online at: https://dissemination-implementation.org/.

[11] Proctor E Silmere H Raghavan R Hovmand P Aarons G Bunger A. Outcomes for implementation research: conceptual distinctions, measurement challenges, and research agenda. Administr Policy Mental Health Mental Health Serv Res. (2010) 38:65–76. 10.1007/s10488-010-0319-7 20957426 PMC3068522

[12] Glasgow RE Harden SM Gaglio B Rabin B Smith ML Porter GC. RE-AIM planning and evaluation framework: adapting to new science and practice with a 20-year review. Front Public Health. (2019) 7:64. 10.3389/fpubh.2019.00064 30984733 PMC6450067

[13] Glasgow RE Battaglia C McCreight M Ayele RA Rabin BA. Making implementation science more rapid: use of the RE-AIM framework for mid-course adaptations across five health services research projects in the Veterans Health Administration. Front Public Health. (2020) 8:194. 10.3389/fpubh.2020.00194 32528921 PMC7266866

[14] Kirk MA Moore JE Wiltsey Stirman S Birken SA. Towards a comprehensive model for understanding adaptations' impact: the model for adaptation design and impact (MADI). Implement Sci. (2020) 15:56. 10.1186/s13012-020-01021-y 32690104 PMC7370455

[15] Wingood GM DiClemente RJ. The ADAPT-ITT model: a novel method of adapting evidence-based HIV interventions. J Acquir Immune Defic Syndr. (2008) 47(Suppl. 1):S40–6. 10.1097/QAI.0b013e3181605df1 18301133

[16] Aschbrenner KA Mueller NM Banerjee S Bartels SJ. Applying an equity lens to characterizing the process and reasons for an adaptation to an evidenced-based practice. Implement Res Pract. (2021) 2:1–8. 10.1177/26334895211017252 34514417 PMC8428660

[17] Aschbrenner KA Bond GR Pratt SI Jue K Williams G Banerjee S. Evaluating agency-led adaptions to an evidence-based lifestyle intervention for adults with serious mental illness. Implement Res Prac. (2020) 1:2633489520943200. 10.1177/2633489520943200 PMC9978662 37089123

[18] Coury J Miech EJ Styer P Petrik AF Coates KE Green BB. What's the “secret sauce”? How implementation variation affects the success of colorectal cancer screening outreach. Implement Sci Commun. (2021) 2:5. 10.1186/s43058-020-00104-7 33431063 PMC7802298

[19] Chambers DA Glasgow RE Stange KC. The dynamic sustainability framework: addressing the paradox of sustainment amid ongoing change. Implement Sci. (2013) 8:117. 10.1186/1748-5908-8-117 24088228 PMC3852739

[20] Skivington K Matthews L Simpson SA Craig P Baird J Blazeby JM. A new framework for developing and evaluating complex interventions: update of Medical Research Council guidance. BMJ. (2021) 374:n2061. 10.1136/bmj.n2061 34593508 PMC8482308

[21] Hawe P Shiell A Riley T. Complex interventions: how “out of control” can a randomised controlled trial be? BMJ. (2004) 328:1561–3. 10.1136/bmj.328.7455.1561 15217878 PMC437159

[22] Perez Jolles M Lengnick-Hall R Mittman BS. Core functions and forms of complex health interventions: a patient-centered medical home illustration. J General Internal Med. (2019) 34:1032–8. 10.1007/s11606-018-4818-7 30623387 PMC6544719

[23] Feldstein AC Glasgow RE. A practical, robust implementation and sustainability model (PRISM) for integrating research findings into practice. Jt Comm J Qual Patient Saf. (2008) 34:228–43. 10.1016/S1553-7250(08)34030-6 18468362

[24] Paniagua-Avila A Fort MP Glasgow RE Gulayin P Hernández-Galdamez D Mansilla K. Evaluating a multicomponent program to improve hypertension control in Guatemala: study protocol for an effectiveness-implementation cluster randomized trial. Trials. (2020) 21:509. 10.1186/s13063-020-04345-8 32517806 PMC7281695

[25] Fort MP Mundo W Paniagua-Avila A Cardona S Figueroa JC Hernández-Galdamez D. Hypertension in Guatemala's public primary care system: a needs assessment using the health system building blocks framework. BMC Health Serv Res. (2021) 21:908. 10.1186/s12913-021-06889-0 34479559 PMC8414027

[26] Hernández-Galdamez D Mansilla K Peralta AL Rodríguez-Szaszdi J Ramírez JM Roche D. Monitoring study participants and implementation with phone calls to support hypertension control during the COVID-19 pandemic: the case of a multicomponent intervention trial in Guatemala. Glob Heart. (2021) 16:77. 10.5334/gh.954 34900568 PMC8622336

[27] Maw AM Morris MA Glasgow RE Barnard J Ho PM Ortiz-Lopez C. Using Iterative RE-AIM to enhance hospitalist adoption of lung ultrasound in the management of patients with COVID-19: an implementation pilot study. Implement Sci Commun. (2022) 3:89. 10.1186/s43058-022-00334-x 35962441 PMC9372925

[28] Palinkas LA Mendon SJ Hamilton AB. Innovations in mixed methods evaluations. Annu Rev Public Health. (2019) 40:423–42. 10.1146/annurev-publhealth-040218-044215 30633710 PMC6501787

[29] Peterson K Anderson J Bourne D Boundy E. Scoping Brief: Care Coordination Theoretical Models and Frameworks. VA Evidence Synthesis Program Evidence Briefs. VA Evidence Synthesis Program Reports. Washington, DC (2011). 30183220

[30] McDonald KM Sundaram V Bravata DM Lewis R Lin N Kraft SA. Closing the Quality Gap: A Critical Analysis of Quality Improvement Strategies, Vol 7. Care Coordination. AHRQ Technical Reviews. Rockville, MD(2007). 20734531

[31] Cordasco KM Hynes DM Mattocks KM Bastian LA Bosworth HB Atkins D. Improving care coordination for veterans within VA and across healthcare systems. J Gen Intern Med. (2019) 34(Suppl. 1):1–3. 10.1007/s11606-019-04999-4 31098970 PMC6542920

[32] Huber TP Rodriguez HP Shortell SM. The influence of leadership facilitation on relational coordination among primary care team members of accountable care organizations. Health Care Manage Rev. (2020) 45:302–10. 10.1097/HMR.0000000000000241 30908316 PMC6755061

[33] Burke RE Kripalani S Vasilevskis EE Schnipper JL. Moving beyond readmission penalties: creating an ideal process to improve transitional care. J Hosp Med. (2013) 8:102–9. 10.1002/jhm.1990 23184714 PMC3650641

[34] McCreight MS Rabin BA Glasgow RE Ayele RA Leonard CA Gilmartin HM. Using the Practical, Robust Implementation and Sustainability Model (PRISM) to qualitatively assess multilevel contextual factors to help plan, implement, evaluate, and disseminate health services programs. Transl Behav Med. (2019) 9:1002–11. 10.1093/tbm/ibz085 31170296

[35] Glasgow RE Fisher L Strycker LA Hessler D Toobert DJ King DK. Minimal intervention needed for change: definition, use, and value for improving health and health research. Transl Behav Med. (2014) 4:26–33. 10.1007/s13142-013-0232-1 24653774 PMC3958586

[36] Emmons KM Viswanath K Colditz GA. The role of transdisciplinary collaboration in translating and disseminating health research: lessons learned and exemplars of success. Am J Prev Med. (2008) 35(2 Suppl.):S204–10. 10.1016/j.amepre.2008.05.009 18619401

[37] Shelton RC Chambers DA Glasgow RE. An extension of RE-AIM to enhance sustainability: addressing dynamic context and promoting health equity over time. Front Public Health. (2020) 8:134. 10.3389/fpubh.2020.00134 32478025 PMC7235159

